# Frequency and Early Complications of Late Preterm Infants: A Descriptive Analysis from Two Secondary-care Hospitals of Karachi

**DOI:** 10.7759/cureus.5789

**Published:** 2019-09-28

**Authors:** Waqar H Khowaja, Abdul Lateef Leghari, Ali Shabbir Hussain, Shabina Ariff, Iqtidar A Khan

**Affiliations:** 1 Pediatrics and Child Health, Aga Khan University Hospital, Karachi, PAK; 2 Pediatrics, Aga Khan University Hospital, Karachi, PAK

**Keywords:** late preterm, early complication, hypoglycemia, hypothermia, sepsis, neonatal jaundice, respiratory distress

## Abstract

Introduction

Globally, prematurity accounts for 12.7% of all live births while late preterm accounts for around three-fourth (73%) of these premature births. In Pakistan, the prevalence of prematurity is approximately 18.89%. Late preterm infants often have weight and size similar to some term infants, but they are still metabolically and physiologically immature. Hence, these infants, as compared to term infants, are at a higher risk of developing medical complications, which results in higher morbidity and mortality during the birth hospitalization. We aim to determine the frequency of early complications in late preterm infants during their stay at Aga Khan Secondary-care Hospitals, Karachi.

Methods

A prospective descriptive study was conducted via the nonprobability sampling technique from March 22, 2016, to March 22, 2017, at secondary-care hospitals of The Aga Khan University Hospital; The Aga Khan Hospital for Women, Karimabad, and The Aga Khan Hospital for Women and Children, Garden. All late-preterm infants, i.e. those born between the 34^0/7^ through 36^6/7^ weeks gestation were included in this study and observed for 72 hours after birth for early complications, including hypothermia, sepsis, hypoglycemia, respiratory distress, and hyperbilirubinemia. Descriptive analysis was done using SPSS Version 19.0 (IBM Corp., Armonk, NY, US) and frequency and percentages were calculated.

Results

Throughout the period of study, a total of 1696 infants were born in secondary-care hospitals, of which 86.67% (n=1470) were term and 13.3% (n=226) were preterm. Late preterm infants constituted 95.5% (n=217) of preterm births and 12.7% of all newborns delivered at study sites. Among them, respiratory distress was diagnosed in 23.5%, hyperbilirubinemia in 17.5%, hypoglycemia in 13.8%, sepsis in 9.2%, and hypothermia in 6%.

Conclusion

Late preterm neonates form the major subgroup of preterm infants delivered at secondary-care hospitals. They have a significant risk of morbidity and birth hospitalizations. We propose that late preterm infants, regardless of their physical dimensions, be given medical attention similar to all preterms.

## Introduction

The most susceptible stage of a child’s survival is characterized by the neonatal phase, i.e. the first month of life. Neonatal mortality poses a major public health issue in many developing countries. Late preterm infants form a large section of preterm births and though considered physiologically immature, the study of the degree of immaturity in these infants has largely remained neglected [1]. Thus, management strategies are deduced from general health-care principles and data about term infants. The difference of physiologic maturation between preterm and term newborns can vary significantly, which also reflects from the biologic differences among infants despite identical gestational ages. Some late preterm infants may physiologically and physically appear like infants born at term, i.e. ≥ 37 weeks’ gestation, whereas most late preterm infants may undergo complications seen in infants born before 34 weeks gestational age, including respiratory distress, apnea, hypothermia, feeding problems, hypoglycemia, hyperbilirubinemia, jaundice, sepsis, and mortality.

The mortality rate of neonates, globally, fell to 19 in 2015 from 36 deaths every thousand live births in 1990, and neonatal deaths declined to 2.7 million from 5.1 million. However, across the globe, in the stretch of 25 years from 1990 to 2015, the decline in neonatal mortality has been slower as compared to under-five post-neonatal mortality (1-59 months) [2]. The similar is true for most low and middle-income countries, including Pakistan, which currently belongs among the five nations accounting for half of the world’s under-five mortality.

If current trends continue, around half of the child deaths (69 million) between 2016 and 2030 will occur in the neonatal age. Currently, 63 countries will have to speed up improvement to achieve the sustainable development goals’ target of mortality rate among neonates of 12 deaths out of one thousand live births by 2030 [3]. Ten countries alone, most of which are in Asia, account for two-thirds of the neonatal deaths that occur across the globe. Third among these countries ranks Pakistan, with more than 500 newborn babies dying every day. With a reported mortality rate of neonates of 42/1000 live births and an estimated total of 298,000 neonatal deaths, 7% of neonatal deaths across the globe occur in Pakistan. Preterm birth is a determining factor in 28% of neonatal deaths worldwide [4-6].

However, in higher-income countries, neonates have gained emphasis in pediatric healthcare so as to reduce morbidity as well as mortality. When compared with third-world countries, however, maternal deaths or deaths among older children <5 years is comparatively given more attention than neonatal mortality rates (NMRs). This also explains why neonatal deaths, keeping in perspective its burden, have not received adequate attention in international community health policy and programs [7]. The greatest influence towards the decrease in neonatal deaths yet achieved has been through the fourth-millennium development goal (MDG), which calls for a two-thirds reduction in mortality risks among under-five children from 1990 to 2015, which, on average, corresponds to a yearly reduction of 4.4% [8]. The literature shows that about 85% of the world’s preterm births occur in Africa and Asia (31% and 54%, respectively), which reflects the disproportionate concentration of burden of preterm births [9-10].

Therapeutic and prophylactic interventions in the last few decades have focused mainly on deliveries before 34 weeks gestation and low birth-weight infants. Many clinicians are now (add negligently) comfortable with births that occur in the late preterm period of gestation while some may even choose elective deliveries before 39-40 weeks of gestation, assuming maturity in terms of metabolism and physiology in these neonates. There is now a growing awareness regarding late preterm births since this group may demonstrate unanticipated complications [11]. “Late-preterm” infants might have the same dimensions in terms of weight and size as some term infants, i.e. those born at 37 to 42 weeks of gestation and thus may be treated by health care professionals, parents, and caregivers as low-risk infants, thus often being managed in newborn level 1 (basic) nurseries or may even remain with their mothers after birth [12].

In comparison to term infants the hospital readmission rates, as well as morbidity and mortality, among these infants are higher due to the complications of prematurity and tend to increase as the gestational age decreases [2].

## Materials and methods

This was a prospective descriptive study conducted at two secondary-care hospitals of The Aga Khan University Hospital from March 22, 2016, for the period of one year. These hospitals, The Aga Khan Hospital for Women, Karimabad, and The Aga Khan Hospital for Women and Children, Garden, were incorporated with The Aga Khan University Hospital (AKUH) in 2010 with the intention to provide access to a quality-based integrated health system to more patients in different areas of Karachi. Today, they provide safe and quality medical care for women and their babies in the hospital. All babies born at these two secondary-care hospitals of The Aga Khan University between the 34^0/7^ through 36^6/7^ weeks gestation were approached and those giving informed consent were included in our study.

A total of 1696 babies were enrolled and observed for 72 hours after birth for early complications, which included signs of hypothermia (temp. <36.50c), neonatal sepsis, hypoglycemia (≈ or less 40 mg/dl), respiratory distress, and hyperbilirubinemia. Neonates transferred out to tertiary care hospitals due to early complications were also included in the study. Final outcomes were measured at the end of 72 hours. Demographic and anthropometric data were collected using medical records. Descriptive analysis was carried out using SPSS version 19.0. Mean and standard deviations were calculated for age. Frequency and percentage were calculated for late preterm delivery and complications such as hypoglycemia, hypothermia, respiratory distress, sepsis, and hyperbilirubinemia. Through stratification of age, effect modifiers were calculated to see the effect of these on the outcome variables. The post-stratification chi-square test was applied, taking p<0.05 as significant.

## Results

**Figure 1:**
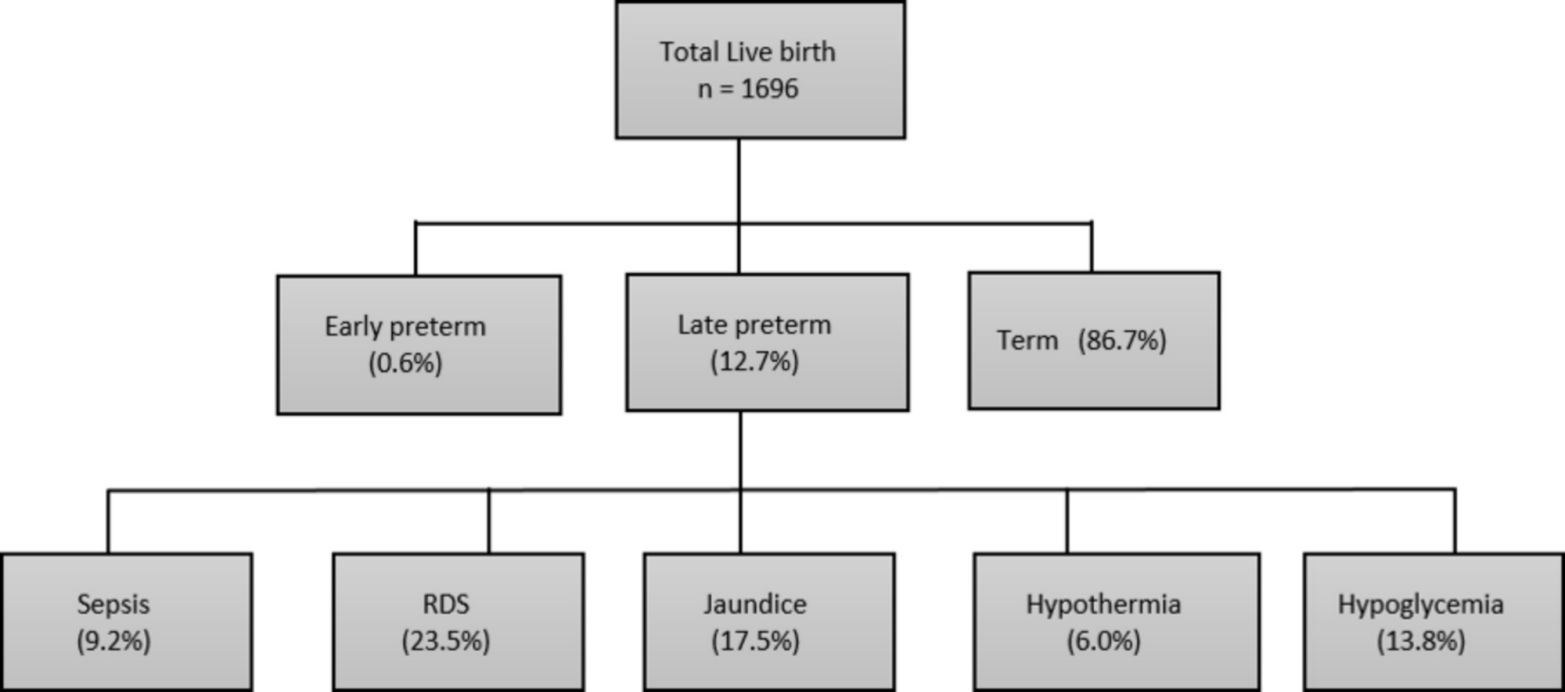
Frequency and early complications of late preterm infants

Throughout the period of the study, an aggregate of 1696 neonates was born in secondary-care hospitals, of which 86.67% (n=1470) were term and 13.3% (n=226) were preterm babies. From the total preterm neonates, 95.5% (n=217) were late preterm, which constitutes 12.7% of all newborn delivered at the study site (Figure 1). In all late preterm neonates, 13.8% (n= 30) were born at 34 weeks, 25.8% (n=56) at 35 weeks, and 60.4% (n=131) at 36 weeks of gestational age. The average birth weight of late preterm infants was 2.56 kilograms ± 0.04 grams.

When morbidities among late preterms were studied, 23.5% (n=51) developed respiratory distress, 17.5% (n=38) developed jaundice, which required phototherapy, 13.8% (n=30) developed hypoglycemia, 9.2% (n=20) of neonates developed sepsis, and 6.0% (n=13) of all late preterm developed hypothermia. An estimation of the risk of morbidities was done with gestational age by applying the chi-square test, taking p<0.05 as significant, which revealed that there is a significant association of hypothermia and respiratory distress with gestational age in late preterm neonates (p-value <0.05), but no association was observed between hypoglycemia, jaundice, and sepsis with gestational age among late preterm in our study (Table 1).

**Table 1:** Early complications of late preterm infants

Gestational Age	34 weeks	35 weeks	36 weeks	P-value
Total Number of Late Preterm	30	56	131	-
Respiratory Distress Syndrome	12	14	25	0.049
Neonatal Jaundice	04	07	27	0.332
Hypothermia	05	02	06	0.028
Hypoglycemia	04	10	16	0.59
Sepsis	03	04	13	0.824

## Discussion

In many countries, the incidence of preterm births is rising largely because of an increase in late preterm births. According to one study conducted in a Texas hospital on 250,000 live-born singletons born late preterm over 18 years, it was found that late-preterm infants accounted for 76% of the preterm infants [13]. In the present study, 13.3% (226/1696) of live-born were preterm out of which 96% were late preterm. Though late preterm comprised the major proportion of prematurity in secondary-care hospitals, they were not being managed as high-risk neonates since their size and weight are very similar to the term neonates and are kept in Level 1 nurseries around the globe.

Great variability has been found in the morbidity and mortality of these neonates among hospitals and among countries, which is also reflective of the quality of neonatal care. The survival, especially of premature infants, tends to be low in developing countries as well as those regions where the facilities in the neonatal intensive care unit (NICU) are limited.

Compared to term infants, late-preterm infants are at greater risk of neonatal morbidity. During the initial birth hospitalization, those who are late preterm are four times more likely to be identified with at least one medical condition as compared to term infants and are three and a half times more likely to be diagnosed with two or more conditions. They are also more likely to have hypoglycemia, respiratory distress, apnea, temperature instability, jaundice, and feeding difficulties, as compared to term infants during the birth hospitalization [1]. In our study, late preterm was assessed during birth hospitalization within 72 hours to record the frequencies of early complications, which showed that the frequency of respiratory distress documented in late preterm is 23.5%, which is common morbidity in this population. A study stated that in total, respiratory morbidities were diagnosed in 13.8% of their population who were late preterm and that with every additional week of gestation, this risk decreased, up to 38 weeks [14]. Similarly, in our study, respiratory distress was significantly associated with gestational age (p<0.05).

Another factor accepted as a major reason for neonatal morbidity and mortality in settings with financial constraints is hypothermia, a common clinical problem observed in the late preterm neonates. In one study, it was reported that 10% of these neonates need active management of hypothermia as compared to those born at term [1]. Our study yielded similar findings, where 6% of neonates developed hypothermia, which was also significantly associated with gestational age (p<0.05).

Since late-preterm infants undergo a delay in development and have a lesser concentration of uridine-diphosphoglucuronate glucuronosyltransferase, therefore, they are more prone to, and suffer for a longer time, from jaundice and hyperbilirubinemia. In 1.2% and 10.7% [14] of term and late preterm infants, respectively, phototherapy was required. Similarly, in this study, phototherapy was required in 17.5% of late preterm neonates. In our study, jaundice too was not significantly associated with gestational age (p>0.05) among the late preterm population.

Sudden loss of maternal glucose and insufficient metabolic response might occur in newborn infants of any gestational age and result in hypoglycemia. In this study, 13.8% of late preterms developed hypoglycemia. Although, the association with gestational age was not statistically significant (p>0.05) among late preterm neonates. Other studies have reported greater incidences of hypoglycemia; around 18% at 35 to 36 weeks of gestation and 4% at term.

Immunological immaturity and relatively poorly developed host defense mechanisms make late preterm infants more prone to septicemia. Whether or not bacterial growth was obtained, evaluation for septicemia was done in 9.2% of late preterm neonates, which had no significant association with gestational age within this population.

## Conclusions

Our analysis exhibits that late preterm birth is a substantial perinatal health issue in our population with regards to short-term morbidities. They are the key subdivision of preterm deliveries at secondary-care hospitals and are at a greater risk of morbidity and birth hospitalization. This subset of the population is at high risk of morbidity and mortality due to multiple factors such as hypothermia, hypoglycemia, sepsis, and respiratory distress. So in order to minimize early neonatal complications in late preterm, the health care provider should give more consideration, regardless of the infant’s size, weight, and physical appearance, as well as early referral to a tertiary care hospital. This evidence-based information will be used to create awareness among health care providers, develop protocols to recognize the complications, and implement timely responses to reduce morbidity and mortality among late preterm neonates.
